# Tensor-Based Learning for Detecting Abnormalities on Digital Mammograms

**DOI:** 10.3390/diagnostics12102389

**Published:** 2022-10-01

**Authors:** Ioannis N. Tzortzis, Agapi Davradou, Ioannis Rallis, Maria Kaselimi, Konstantinos Makantasis, Anastasios Doulamis, Nikolaos Doulamis

**Affiliations:** 1 Department of Rural Surveying Engineering and Geoinformatics Engineering, National Technical University of Athens, 157 80 Athens, Greece; 2 Department of Artificial Intelligence, University of Malta, MSD 2080 Msida, Malta

**Keywords:** mammography, deep learning, machine learning, tensor-based learning, CP decomposition, breast cancer, computer-aided detection, screening

## Abstract

In this study, we propose a tensor-based learning model to efficiently detect abnormalities on digital mammograms. Due to the fact that the availability of medical data is limited and often restricted by GDPR (general data protection regulation) compliance, the need for more sophisticated and less data-hungry approaches is urgent. Accordingly, our proposed artificial intelligence framework utilizes the canonical polyadic decomposition to decrease the trainable parameters of the wrapped Rank-R FNN model, leading to efficient learning using small amounts of data. Our model was evaluated on the open source digital mammographic database INBreast and compared with state-of-the-art models in this domain. The experimental results show that the proposed solution performs well in comparison with the other deep learning models, such as AlexNet and SqueezeNet, achieving 90% ± 4% accuracy and an F1 score of 84% ± 5%. Additionally, our framework tends to attain more robust performance with small numbers of data and is computationally lighter for inference purposes, due to the small number of trainable parameters.

## 1. Introduction

Breast cancer is the leading cause of death in women worldwide, accounting for more than 685,000 deaths in 2020, and it is the most commonly diagnosed type of cancer, with more than 2.26 million new cases [1]. It is a variation of malignant growth expanding from breast tissue, often in the interior area of the breast, metastasizing to other body areas (i.e., lymph nodes). It commonly affects women above 40 years old, with the main risk factors being the patient’s age, family history, and level of obesity [2,3]. Fortunately, observational studies have shown that the early-stage detection of breast nodules leads to a very high 5-year survival rate, exceeding 90%, while on the contrary, the survival rate drops by 27% in cases of late diagnosis [4]. This emphasizes the need for better prognosis and the development of improved screening strategies.

The assessment of breast cancer detection in a non-invasive manner is very important for identifying abnormal regions of interest (ROIs) on medical imaging modalities. One of the most effective non-invasive screening techniques for the early detection of breast cancer is digital mammography. It is the most commonly used diagnostic test; it uses low-energy X-rays to identify lumps in dense tissue and has been proven to assist in the decrease in mortality rates [5,6]. However, despite its advantages, mammography presents many limitations. More specifically, it is associated with (a) high risk of false positives [7,8,9], where in many cases the biopsy detects no cancer, as well as (b) a high risk of false negatives [10,11,12], where the breast cancer remains underdiagnosed. Therefore, in recent decades, many methods have been adopted in order to help radiologists reduce the diagnostic errors of screening mammography while avoiding invasive exams (i.e., needle biopsy) [13].

The rapid growth of artificial intelligence (AI) provides robust tools for helping health-care experts to identify and classify potential tumors and calcifications and reduce the mammographic screening reading workload [14]. In more detail, a lot of effort has been put on applying AI techniques on low-cost diagnosis solutions such as mammography [15], lung segmentation [16], and other medical imaging applications [17]. The improvement, however, of the existing AI algorithms [18] is hindered by limitations of data availability, which is considered a major drawback. This occurs from two major factors; first, the lack of publicly available large datasets and, second, the requirement of many AI framework architectures (i.e., federated learning [19] in an effort to address GDPR compliance) dictating the training procedure to be applied on small sets of data.

Based on the discussion above, in this study we introduce the idea of tensor-based learning for the automatic mammography reading. Tensor-based learning allows us to efficiently address small sample setting problems, where the number of data for training the models is limited, without compromising the models’ prediction accuracy. To achieve this, the canonical polyadic (CP) decomposition of rank *R* is applied on the proposed model’s trainable parameters to significantly reduce their number. Hence, we name the proposed model Rank-*R* Fully-connected Neural Network (FNN). The proposed Rank-*R* FNN is capable of characterizing tissue in mammography images by exploiting the structural information of the input. According to the experimental results on the open digital mammographic database INBreast, our solution outperforms competitive deep learning methods, and at the same time, it is more efficient in terms the amount of training data required, as well as the computational cost for inference purposes. On the contrary, the proposed model presents some limitations mostly related to the additional pre-processing effort needed and the higher computation time for the training process.

The remainder of the paper is structured as follows: Section 2 presents related works on applying deep learning models and, specifically, convolutional neural networks (CNNs) on mammography screening for breast tumor classificication. Section 3 describes the proposed tensor-based learning system for mammogram classifications, as well as the applied pre-processing steps. In Section 4, an extensive experimental evaluation of the discussed methods is provided, while Section 5 provides a summary of findings and concluding remarks.

## 2. Related Work

Many attempts have been carried out focusing on the identification of malignant areas and the classification of tumors. Many researchers have focused on applying modern deep learning architectures based on CNNs [20] for detecting and classifying breast cancer. Below, we present a few such indicative works.

The authors in [21] propose an ensemble approach for breast neoplasm classification as benign or malignant, by combining mammogram imaging and spectral signals of blood plasma samples. Their proposed solution utilizes a recurrent neural network (RNN) for processing spectral signals and the deep CNN AlexNet [22] for image processing. The processed images and spectral signals are fused into a common representation, which is then fed into a support vector machine (SVM) responsible for classifying neoplasm as benign or malignant. In the work of [23], a new framework for segmentation and classification of breast cancer images is presented. More specifically, the proposed technique is based on different deep learning models, including InceptionV3 [24,25], DenseNet121 [26], ResNet50 [27], VGG16 [28], and MobileNetV2 [29], for the classification task, and a modified U-Net for the breast segmentation. The framework was evaluated on three mammographic datasets and the modified U-Net model [30] alongside the InceptionV3 model, which achieves the best result. In the study of [31], a fusion model is developed that utilizes the you-only-look-once (YOLO) architecture [32] to localize and classify abnormalities on digital mammograms. The proposed method was evaluated on both current, as well as original and synthetic, prior mammograms and identifies three different types of lesions: mass, calcification, and architectural distortions. In more detail, the CycleGAN [33] and Pix2Pix [34] techniques were used to generate the new translated prior mammograms, which resemble the current mammograms, while preserving the general texture of the prior ones. The study in [35] introduced the CoroNet model, which is based on the Xception CNN architecture [36] and is capable of performing automatic four-class (benign mass/malignant mass and benign calcification/malignant calcification) and two-class (calcifications and masses) classification of breast cancer. The presented model is pre-trained on the ImageNet dataset and fully trained on mammograms. In [37], the authors present a residual-aided classification U-Net model (ResCU-Net) for simultaneous mass segmentation and classification. The proposed model incorporates the U-Net and SegNet [38] architecture aided by residual blocks to exploit multilevel information for achieving improved tissue identification. The study in [39] proposes a multi-view feature fusion network model for classification of mammography images in two stages (normal/abnormal and benign/malignancy), based on multi-scale attention DenseNet. Their work mainly focuses on the construction of the multi-scale convolution module, which uses convolution kernels of different scales for image feature-extraction, as well as the construction of the attention module, which connects to a parallel channel attention module and a spatial attention module.

Besides the exploitation of established deep learning architectures, many researchers have relied on more custom CNN architectures. Indicatively, in [40], the deep-learning-assisted efficient adaboost algorithm (DLA-EABA) is proposed. The suggested solution utilizes the AdaBoost algorithm for the final prediction function, alongside a CNN to characterize breast masses in several imaging modalities, such as magnetic resonance imaging (MRI), ultrasound (US), digital breast tomosynthesis, and mammography. In [41], the authors present a Multiscale All CNN (MA-CNN) to automatically categorize the mammogram images into normal, malignant, and benign classes. The MA-CNN model achieves higher classification accuracy by fusing the wider context of information using multiscale filters without affecting the computation speed. In [42], a method for the automatic detection and classification of cancerous regions in mammograms is presented, in which a CNN, alongside the grasshopper optimization algorithm (GOA) [43], is utilized. The GOA-based CNN achieves optimized feature extraction and feature selection, as well as decreased computational cost.

Although all the aforementioned CNN-based approaches are cost-effective solutions with increased predictive accuracy, they need a huge number of annotated data to be efficiently trained, which is still lacking at the required scale [44]. This makes them inapplicable to be used as medical imaging solutions and raises the need for the further development of low-cost and lightweight systems that tackle the shortcoming of a lack of medical data.

### Our Contribution

The importance of our work is summarized in three major pillars:

- The creation of small sets for training purposes, in an effort to meet real-world criteria meaning the limited number of data;
- The utilization of CP decomposition to reduce the number of data needed for the training of the proposed Rank-R FNN model; and
- The requirement of lower computational cost due to the lower amount of trainable parameters.

The employment of the filters converts the initial two-dimensional images to three-dimensional objects, enriching the raw information with additional low-level image features. It should be noted that the employment of the aforementioned filters takes place without requiring any training or parameter tuning. Accordingly, the tensor-based model exploits these auxiliary features and combines it with further spatial information extracted from the neighborhood of the pixel under examination. The lower computational cost comes straight from the reduction in the trainable parameters. In this way, the proposed solution is proved to be a robust tool for classifying ROIs on digital mammograms.

## 3. Methodology

In this section, we first formulate the problem of automatic detection of abnormalities on digital mammographies, and then we present the proposed Rank-*R* FNN for tackling that problem.

### 3.1. Problem Formulation

The problem of the automatic detection of abnormalities on digital mammographies can be seen as a classification problem, where the objective is to classify every mammography pixel to one out of *C* available classes (e.g., healthy, calcification, and malignant classes). A mammography pixel at location (x,y) on the image plane is represented by a scalar value or by a set of values depending on the number of image channels. Using that information to classify a pixel will result in a classifier that disregards the pixel’s spatial information, that is, the relationship of the pixel at (x,y) with its neighboring pixels. To incorporate spatial information into the classifier, we represent a pixel at location (x,y) with the values of a patch of pixels centered at the same location. Then, the pixel classification problem is transformed into a patch classification problem, where the class of the pixel at location (x,y) is the same as the class of the patch centered at (x,y). This approach also followed in [45,46,47] yields more robust classification models. Based on the discussion above, we describe below the formulation for the problem of automatic detection of abnormalities on digital mammograms.

Given a set *X* of *N* patches, we want to map each element $X_{i}\left(i=1,\ldots ,N\right)$ to one of the available classes. Let *C* denote the number of these classes and $t_{i}$ represent the ground truth label vector for the corresponding $X_{i}$ patch. The vector $t_{i}$ consists of C−1 zero-value elements and a single element with a value equal to 1, which depict the class to which the $X_{i}$ patch belongs. Alternatively, $t_{i}={\left[t_{i,1},t_{i,2},\ldots ,t_{i,C}\right]}^{T}\in {\left\{0,1\right\}}^{C}$, given that $\sum_{c=1}^{C}t_{i,c}=1$. Eventually, these pairs $\left(X_{i},t_{i}\right)$ compose the final dataset *D* that is used to feed the models for training and testing purposes. *D* is defined mathematically by Equation (1).

(1) $$D={\left\{\left(X_{i},t_{i}\right)\right\}}_{i=1}^{N}$$

The goal is to create a function *f* that is formed by a set of parameters θ∈Θ and correctly predicts the class of a given patch $X_{i}$. The output of *f* would be a vector containing the estimated probabilities for $X_{i}$ to belong to each class. Thus,

(2) $$f\left(X_{i},\theta \right)=\left[p^{1}\left(X_{i},\theta \right),\ldots ,p^{k}\left(X_{i},\theta \right),\ldots ,p^{C}\left(X_{i},\theta \right)\right]$$

where $p^{k}\left(X_{i}|\theta \right)$ shows the conditional probability that the *i*-th sample belongs to $k_{th}$ class given $X_{i}$ and the parameters θ. The final prediction of the class is given by

(3) $$t_{i}^{*}=\arg\max_{1\leq k\leq C}p^{k}\left(X_{i},\theta \right),$$

which replaces all the values with zeros in the output vector, except for the element with the highest probability, which is set to 1.

To create a proper function, we have to minimize the number of cases where the predictions are different than the corresponding ground truth labels, that is, $t_{i}^{*}\neq t_{i}$. This objective is directly related to the estimation of parameters θ, so that

(4) $$\theta^{*}=\arg\min_{\theta \in \Theta }\frac{1}{N}\sum_{i=1}^{N}L_{CE}\left(f\left(X_{i},\theta \right),t_{i}\right)$$

where $L_{CE}$ refers to the cross-entropy loss function and θ∈Θ is the set of parameters that defines the form of *f*.

### 3.2. Rank-*R* FNN Model for the Automatic Detection of Abnormalities in Mammograms

As mentioned in the previous section, we represent each mammography pixel *i* by a square patch of pixels centered at the *i*-th pixel’s location. This way, we are able to exploit the pixel’s spatial information encoded in its neighboring pixels. Therefore, each pixel *i* is represented by a third order tensor $X_{i}\in R^{s\times s\times b}$, where *s* stands for the height and width of the patch and *b* for image channels.

To address the problem formulated in the previous section, we represent the function *f* by a Rank-*R* FNN model model. The Rank-*R* FNN model is a neural network with one hidden layer that consists of, let us say, *Q* hidden neurons. Rank-*R* FNN weights connecting the input to hidden layer are tensors satisfying the Rank-*R* canonical polyadic decomposition [48]:

(5) $$w^{\left(q\right)}=\sum_{k=1}^{R}w_{3,k}^{\left(q\right)}\circ w_{2,k}^{\left(q\right)}\circ w_{1,k}^{\left(q\right)}\in R^{b\times s\times s},$$

for q=1,⋯,Q with $w_{3,k}^{\left(q\right)}\in R^{b}$ and $w_{i,k}^{\left(q\right)}\in R^{s}$, i=1,2. Superscript *q* denotes that these weights connect the input to the *q*-th neuron of the hidden layer, and “∘” operator stands for vectors outer product. The output of the Rank-*R* FNN for the *i*-th sample and *c*-th class is

(6) $$p_{i}^{c}=\sigma \left(\langle v^{\left(c\right)},u_{i}\rangle \right),$$

where $v^{\left(c\right)}$ collects the weights between the hidden layer and the *c*-th output neuron, σ(·) denotes the softmax activation function, and $u_{i}={\left[u_{i,1},u_{i,2},\cdots ,u_{i,Q}\right]}^{T}$ with

(7) $$u_{i,q}=g\left(\langle \left(\sum_{k=1}^{R}w_{3,k}^{\left(q\right)}\circ w_{2,k}^{\left(q\right)}\circ w_{1,k}^{\left(q\right)}\right),X_{i}\rangle \right)$$

for q=1,⋯,Q to be the output of the hidden layer activated by function g(·). Given a collection of training data in the form of relation (1), we estimate the set of parameters of the employed models using the backpropagation algorithm [49] with the Adam gradient based optimizer [50]. In the case of Rank-*R* FNN, the parameters θ of function *f* are the set $\left\{w^{\left(q\right)},v^{\left(c\right)}\right\}$ for q=1,⋯,Q, and c=1,⋯,C.

## 4. Dataset and Pre-Processing

### 4.1. Dataset Description

For the purposes of this study, the INBreast dataset [51] was utilized. It is a collection of 410 mammograms that corresponds to 115 independent cases, 90 of which refer to women with both breasts affected, while the other 25 women have undergone mastectomy. Additional information, such as the BIRADS score, the density level, the existence of tumor or calcification, and other indexes, are included in an auxiliary CSV file. Segmentation masks, containing precise contouring of potential tumors or calcification, are provided in XML format for each non-healthy image.

In this paper, we do not take into account the grouping according to the patient each mammogram belongs to. Instead, we consider each image as a standalone object that comes with further information: (a) the lesion existence binary index, (b) the calcification existence binary index, and (c) the segmentation contouring details.

### 4.2. Pre-Processing Pipeline

**Figure 1:**
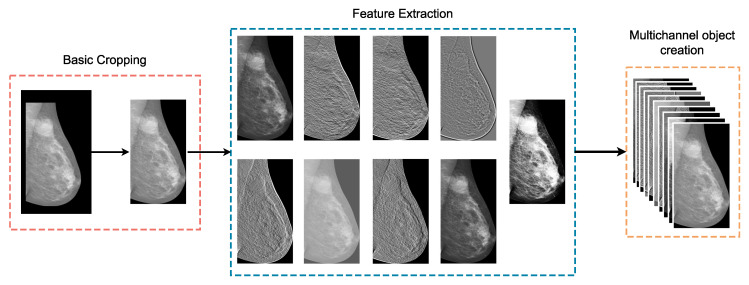
This figure presents the first stage of the proposed AI framework, where peripheral cropping is applied on the input image and low-level features are extracted using digital filters.

The first major task of the pre-processing pipeline is related to the enrichment of the given images using some basic low level filters aiming to exploit any potential features related to the ROIs. Accordingly, we utilized the following filters: Sobel in combination with different threshold values, the Canny edge detector, Gaussian difference, gamma correction, histogram normalization, and Gabor. As shown in Figure 1, a basic cropping procedure is applied on the initial mammography, nine independent filters are derived from the cropped image, and a multichannel object is produced, including the raw image. In this way, the initial mammogram is transformed to a three-dimensional object that contains more information and additional features to be exploited.

### 4.3. Extraction of Patches

**Figure 2:**
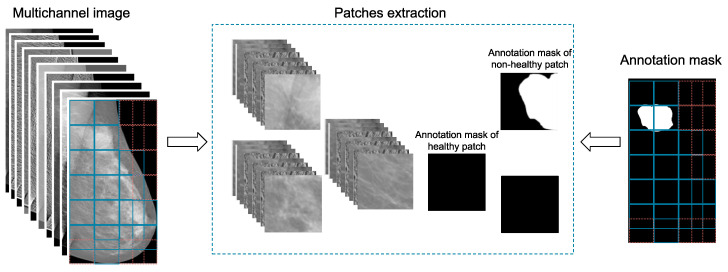
This figure presents the second stage of the proposed AI framework, where patches are retrieved from the enriched, multichannel image object. The relevant areas are extracted from the corresponding annotation mask.

The initial mammogram contains meaningless information, such as areas with no breast tissue. A peripheral cropping technique is applied to eliminate part of this area as shown in Figure 1. However, it still remains a significant part of the image that consists of unwanted details. Thus, the idea of patch extraction, shown in Figure 2, is adopted. According to this approach, the image is traversed horizontally, using a predefined step, and only patches that satisfy a set of criteria are extracted and stored for further processing. These criteria are (a) the predefined number of patches to be extracted by a single image; (b) the coverage of breast tissue inside a patch should exceed 90% of the patch’s area; and (c) the inclusion of ROIs or part of them in the patch, if the image contains any type of lesion.

### 4.4. Tensorization

**Figure 3:**
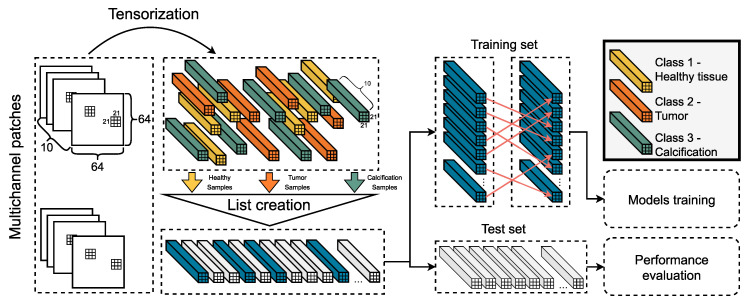
This figures depicts the third stage of the proposed AI framework. The selected patches are transformed into tensor objects, which are then stored in a temporary list. After a sampling process, the training and the testing sets are formed. A permutation is applied to the former and both of them are fed to the AI models.

The tensorization technique parses a given patch of the image and creates a tensor object for each pixel, which we call dominant pixel. The size of this object depends on the tensor window size (TWS) hyper-parameter, exploiting the additional spatial information of the neighborhood. The class of the tensor object is the same as the one of the dominant pixel, as depicted in the annotation mask of the corresponding patch. The tensorization process is depicted as the initial step of the pipeline in Figure 3.

### 4.5. Final Dataset Preparation

When the tensorization procedure is completed, all the tensor objects that occur are stored in a temporary list. Based on the samples per class (SPC) hyper-parameter, the sampling component picks the samples that will be used for the construction of the training set, while the rest of them are left for framing the testing set. Aiming to ensure an unbiased training process, a permutation process is applied on the training set. Both sets are fed into the available deep learning models, and the results are combined in proper diagrams to evaluate their performance. To meet real-world criteria, meaning the limited number of data, mini sets for training purposes are constructed.

### 4.6. The Pipeline in a Nutshell

From the 115 total cases, only the 90 of them, which refer to women with both breasts affected, are taken into account for the purposes of this work. These 90 cases correspond to 360 mammograms, considering two views (MLO and CC) of each breast for all cases. The mammograms are processed as standalone images, which means that no conceptual interconnections among them are taken into consideration (i.e., two images depict the same breast from different view, two images correspond to the same case etc). The low-level features occur from the digital filters’ application on the original image, which are combined, along with the original image, in a single three-dimensional object. This initial pre-processing step is described in Section 4.1 and Section 4.2 and depicted in Figure 1.

In the second stage, we automatically extract patches of size 64×64×10 pixels from each multichannel object, in a manner that no useless information is included. This stage of the pipeline is analyzed in Section 4.3 and presented in Figure 2.

In the final stage, the extracted patches are split into tensors of size TWS × TWS × 10. The SPC hyper-parameter defines the amount of tensors to be extracted from each patch. The SPC values are selected in such a way that a small dataset is constructed, to create proof of concept scenarios; a small number of data are needed for the training process. In Figure 3, we describe, as an example, the process for splitting the 64×64×10 patches into tensors with size 21×21×10. As shown in Figure 3, each class is sufficiently represented in the final dataset (based on the SPC hyper-parameter), and the tensor samples are illustrated in the figure with different colors. Though, since the tensors’ size is small enough (TWS × TWS × 10), we end up with many tensors and an accordingly a long dataset. In an effort to address this issue, we assign 30% of this dataset to be used for the training process and the rest of it (70%) to be used for the validation purposes. This final stage is described in Section 4.4 and Section 4.5 and depicted in Figure 3.

Every time the experiment is repeated, the tensors are selected randomly from the extracted patches. Thus, we conducted all the experiments several times. In this way, we ensure that all the methods, including the proposed one, are evaluated on the majority of the information provided in the original INBreast dataset.

## 5. Experimental Validation

We compare the proposed tensor-based architecture against state-of-the-art deep learning models for detecting abnormalities on digital mammograms. In particular, we compare it with (a) a fine-tuned version of the CNN model presented in [52]; (b) the model used in [53] inspired by the AlexNet architecture [22]; (c) an improved CNN model architecture combined with a UNet model adopted in [54]; and (d) the model proposed in [55], which is based on the SqueezeNet approach [56]. All models were adapted to fit our dataset and were fine-tuned to achieve higher performance. We designed a series of experiments based on the tuning of the hyper-parameters, presented in Table 1, that are common for both models. 64×64 patch size was selected for eliminating the areas with useless information and retrieving patches that include satisfying regions of interest. In all experiments, the models were trained for 70 epochs, and a validation process was applied on the testing set every 10 epochs. Each distinct experiment was repeated 10 times to ensure the convergence of the results and report statistics.

**Figure 4:**
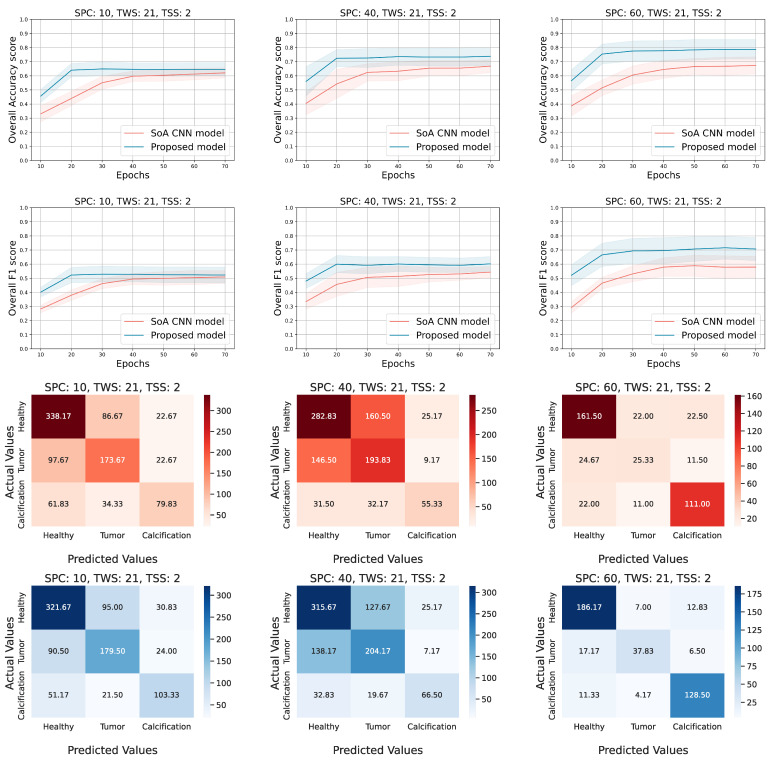
Experimental results for the following configuration; TWS = 21, SPC = (10, 40, and 60) and TSS = 2. The overall accuracy and F1 scores on the testing set are presented in the first two rows respectively. The rest of the rows correspond to the confusion matrices of the CNN (red hues) compared to our approach (tensor-based model (blue hues)).

**Figure 5:**
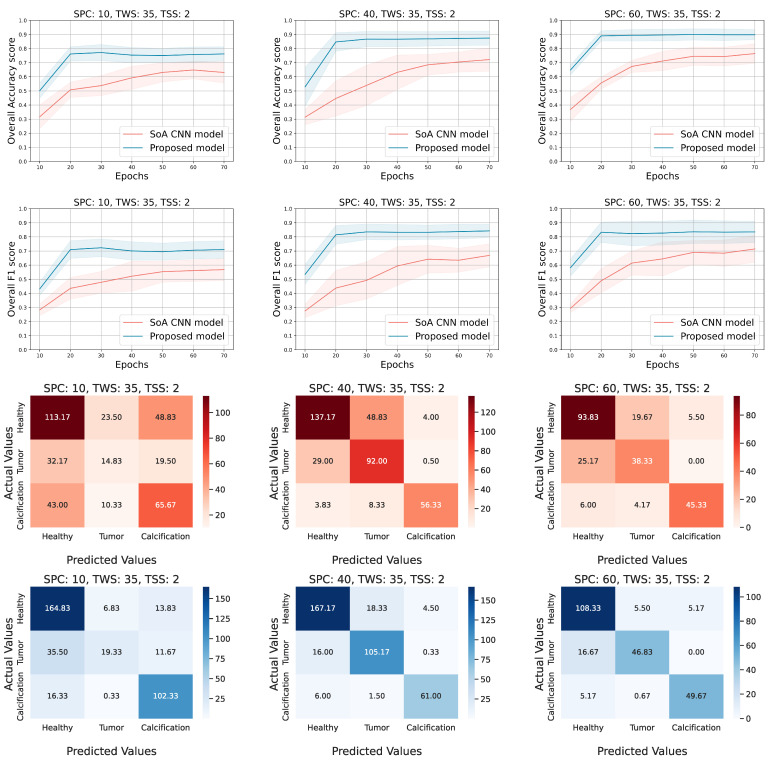
Experimental results for the following configuration; TWS = 35, SPC = (10, 40, and 60) and TSS = 2. The overall accuracy and F1 score on the testing set are presented in the first two rows, respectively. The rest of the rows correspond to the confusion matrices of the AlexNet (red hues) and the tensor-based approach (blue hues) accordingly.

In Table 2, we present the mean accuracy and F1 scores, calculated on the testing set, for all the models and all the experimental configurations. The first two columns refer to the configuration of each experiment, the third column describes the metrics, and the rest of the columns present the performance of the several models from the perspective of each metric. Each value represents the mean score achieved by the corresponding model and is followed by the 95% confidence interval occur by the repetition of the experiments. In most cases, our proposed approach achieves higher performance than the other solutions. Furthermore, the smaller range of the 95% confidence intervals in our proposed solution confirms that the tensor-based model tends to be more robust and stable. However, there are some overlaps that appeared between the 95% confidence intervals for the different models. We discuss in detail this overlap in the next paragraphs, where we describe the Figure 4 and Figure 5.

After performing all the experiments for the aforementioned state-of-the-art deep learning models, we selected the AlexNet [22] to extract additional metrics and compare it side by side with the proposed approach. Thus, Figure 4 and Figure 5 show the 95% confidence interval of the average accuracy and F1 metrics across the epochs and the corresponding confusion matrices. Specifically, they present the overall accuracy and F1 score of both models, over the testing set, for different combinations of TWS and SPC. Two distinct scenarios occur; (a) keep TWS constant and increase the SPC, (b) keep SPC constant and increase the TWS. The TSS value is set small enough to exploit the spatial correlation of the ROI pixels by maintaining the overlapping areas among the extracted tensor objects. Conforming to our initial concept, tensor-based learning should perform better when the samples are limited and the window size is large enough in order for the spatial information to be utilized. Thus, for each TWS value we chose three different and low values of SPC to observe the behavior of both models in small amount of samples.

Evaluating the performance of the models when TWS = 21 (Figure 4), we observe that the tensor-based model performs better in terms of the accuracy score for all values of SPC (10, 40, 60). Similar outcomes for the F1 score, even though sometimes the AlexNet model achieves lower deviation from the mean value. It is noticed that both models present low performance when the SPC is set to 10 (10 samples from each patch of the dataset). It is clear that the low number of data, in combination with the small window size of the tensors (TWS), provide limited information that is not sufficient for the training process of the models. On the other hand, when either the SPC or the TWS (Figure 5) is set to a higher value, the performance metrics are higher and the proposed approach seems to be the superior one. The confusion matrices of both models (red hues for AlexNet and blue hues for tensor-based approach) ensure that the overall accuracy score corresponds to all the three classes. It is remarkable that the proposed model tends to be more accurate in all classes; in the majority of the cases, the diagonal values are higher than the corresponding ones of the AlexNet approach, while the non-diagonal values are lower.

Evaluating the performance of the models when TWS = 35 (Figure 5), it is shown that the proposed model presents higher accuracy and F1 scores, while it converges smoothly and has a more robust behavior in contrast to the AlexNet approach. Additionally, the proposed model reaches the highest performance fast (after 25–30 epochs of training) and achieves almost 93% accuracy in some configurations. Moreover, the confusion matrices confirm that the tensor-based model performance is well distributed among the several classes. At the same time, the AlexNet is characterized by higher confusion, especially for the first two classes, in comparison with the proposed solution.

In a few cases, it was observed that some of the state-of-the-art models performed better than the proposed method. The third row in Table 2, for example, presents the experiment with SPC = 60 and TWS = 35; the proposed approach presents a mean accuracy score equal to 90% with a variation of 4% through the several repetitions, while the model presented in [53] achieves mean accuracy score equal to 78% with a variation 8% for the same configuration. This means that there are few repetitions of the experiment where the state-of-the-art model performs better from the perspective of an accuracy score. Such situations are observed either due to specific configuration of the experiment parameters or due to irregularities of the final dataset through the several repetitions of a particular experiment.

The accuracy and f1 score curves in Figure 4 show that the models under comparison are close enough and the overlap is dense when the SPC is low, while their gap gets reduced and the overlap is more sparse as the SPC increases for both metrics. On the contrary, the Figure 5 shows that the minimum gap between the two models seems to be almost constant as the SPC increases, and the corresponding overlap is slight in a couple of cases. In the first case, where TWS = 21, it is obvious that the proposed method does not perform well when both TWS and SPC are low and the overlap is dense. In the second case, where TWS = 35, the proposed method performs well even for low values of SPC, and the overlap is sparse where it exists.

## 6. Conclusions

In this work, we introduce a tensor-based learning model for the classification of mammogram images. Our solution uses a reduced number of trainable parameters of the wrapped Rank-R FNN model by utilizing the canonical polyadic decomposition, which leads to an improved training process with fewer data. The proposed AI framework is evaluated on the INBreast dataset and compared against some state-of-the-art models such as a CNN model, an AlexNet implementation, a ConvNeXt approach, and a SqueezeNet model.

The experimental results demonstrate that the tensor-based model presents better mean performance in comparison with the aforementioned models for the most tested configurations of small numbers of training data as it achieves higher accuracy and F1 scores. In addition, our proposed model presents lower deviation and requires fewer epochs for training in the majority of the experimental tests, while most of the others show more unstable training. In addition, we concluded that the most proper TWS value equals 35 since, in this configuration, the high accuracy scores correspond to the most precise detection of the distinct classes as well.

In general, according to the worst-case scenario, the proposed model prevails over the state-of-the-art approaches about 2–5% of the time, while according to the best case scenario the proposed model can achieve 20% higher accuracy. Finally, our study demonstrates that the presented tensor-based learning model can be sufficiently applied on medical data and achieves accurate results in cases with limited data. On the other hand, the developed tensor-based framework requires plenty of pre-processing actions such as the creation of multichannel objects, the extraction of patches and the tensorization procedure, and higher computation time for training. Such negative aspects of the presented approach constitute a significant challenge for us given that we aim to optimize the processes and implement an integrated solution.

## Figures and Tables

**Table 1:** The hyper-parameters of the proposed framework.

Name	Description	Value Range	Units
TWS	Tensor window size	>3	pixels
SPC	Samples per class	≥10	samples
SPS	Selected patch size	32–512	pixels
TSS	Tensor step size	≥1	pixels

**Table 2:** Comparison of the performance metrics (accuracy and F1-score) between the proposed approach and state-of-the-art deep learning models for detecting abnormalities in mammograms. Each value represents the mean score achieved for different experimental configurations (SPC and TWS) followed by the 95% confidence interval.

Samples Per Class (SPC)	Tensor Window Size (TWS)	Metrics	Our Proposed Tensor-Based Model	Desai and Shah, 2021 [52]	Mohapatra et al., 2022 [53]	Han et al., 2022 [54]	Saxena et al., 2020 [55]
10	35		77%±5%	65%±7%	65%±6%	70%±3%	73%±3%
40	35		88%±5%	78%±9%	72%±8%	69%±12%	74%±10%
60	35	**Testing set**	90%±4%	76%±7%	78%±8%	81%±9%	75%±15%
10	21	**Accuracy**	65%±5%	63%±6%	62%±3%	54%±5%	60%±6%
40	21		73%±7%	68%±6%	68%±4%	70%±10%	70%±13%
60	21		79%±7%	65%±6%	68%±5%	60%±15%	65%±15%
10	35		71%±6%	56%±5%	58%±8%	55%±4%	68%±10%
40	35		84%±5%	72%±9%	68%±8%	70%±10%	65%±20%
60	35	**Testing set**	83%±9%	73%±7%	71%±10%	80%±12%	65%±20%
10	21	**F1**	52%±6%	50%±3%	51%±4%	45%±5%	46%±15%
40	21		60%±5%	55%±5%	55%±5%	55%±6%	59%±12%
60	21		71%±9%	55%±6%	69%±8%	58%±12%	52%±16%

## Data Availability

The final dataset was constructed by splitting the original images of the open-source INBreast dataset into patches and tensors as described in Section 4.

## References

[1] Ferlay J., Colombet M., Soerjomataram I., Parkin D.M., Piñeros M., Znaor A., Bray F. (2021). Cancer statistics for the year 2020: An overview. Int. J. Cancer.

[2] Colditz G.A., Kaphingst K.A., Hankinson S.E., Rosner B. (2012). Family history and risk of breast cancer: Nurses’ health study. Breast Cancer Res. Treat..

[3] Alegre M.M., Knowles M.H., Robison R.A., O’Neill K.L. (2013). Mechanics behind breast cancer prevention-focus on obesity, exercise and dietary fat. Asian Pac. J. Cancer Prev..

[4] DeSantis C.E., Ma J., Gaudet M.M., Newman L.A., Miller K.D., Goding Sauer A., Jemal A., Siegel R.L. (2019). Breast cancer statistics, 2019. CA A Cancer J. Clin..

[5] Lee C.S., Monticciolo D.L., Moy L. (2020). Screening guidelines update for average-risk and high-risk women. Am. J. Roentgenol..

[6] Oeffinger K.C., Fontham E.T., Etzioni R., Herzig A., Michaelson J.S., Shih Y.C.T., Walter L.C., Church T.R., Flowers C.R., LaMonte S.J. (2015). Breast cancer screening for women at average risk: 2015 guideline update from the American Cancer Society. JAMA.

[7] Berg W., Hendrick E., Kopans D., Smith R. (2009). Frequently Asked Questions about Mammography and the USPSTF Recommendations: A Guide for Practitioners. Rest. Soc. Breast Imaging.

[8] Lehman C.D., Arao R.F., Sprague B.L., Lee J.M., Buist D.S., Kerlikowske K., Henderson L.M., Onega T., Tosteson A.N., Rauscher G.H. (2017). National performance benchmarks for modern screening digital mammography: Update from the Breast Cancer Surveillance Consortium. Radiology.

[9] Hofvind S., Ponti A., Patnick J., Ascunce N., Njor S., Broeders M., Giordano L., Frigerio A., Törnberg S. (2012). False-positive results in mammographic screening for breast cancer in Europe: A literature review and survey of service screening programmes. J. Med. Screen..

[10] Kuhl C.K. (2015). The changing world of breast cancer: A radiologist’s perspective. Investig. Radiol..

[11] Karssemeijer N., Bluekens A.M., Beijerinck D., Deurenberg J.J., Beekman M., Visser R., van Engen R., Bartels-Kortland A., Broeders M.J. (2009). Breast cancer screening results 5 years after introduction of digital mammography in a population-based screening program. Radiology.

[12] Bae M.S., Moon W.K., Chang J.M., Koo H.R., Kim W.H., Cho N., Yi A., La Yun B., Lee S.H., Kim M.Y. (2014). Breast cancer detected with screening US: Reasons for nondetection at mammography. Radiology.

[13] Tran W.T., Sadeghi-Naini A., Lu F.I., Gandhi S., Meti N., Brackstone M., Rakovitch E., Curpen B. (2021). Computational radiology in breast cancer screening and diagnosis using artificial intelligence. Can. Assoc. Radiol. J..

[14] Rodriguez-Ruiz A., Lång K., Gubern-Merida A., Teuwen J., Broeders M., Gennaro G., Clauser P., Helbich T.H., Chevalier M., Mertelmeier T. (2019). Can we reduce the workload of mammographic screening by automatic identification of normal exams with artificial intelligence? A feasibility study. Eur. Radiol..

[15] Wu N., Phang J., Park J., Shen Y., Huang Z., Zorin M., Jastrzębski S., Févry T., Katsnelson J., Kim E. (2019). Deep neural networks improve radiologists’ performance in breast cancer screening. IEEE Trans. Med Imaging.

[16] Ali R., Hardie R.C., Ragb H.K. Ensemble lung segmentation system using deep neural networks. Proceedings of the 2020 IEEE Applied Imagery Pattern Recognition Workshop (AIPR).

[17] Ali R., Hardie R.C., Narayanan B.N., Kebede T.M. (2022). IMNets: Deep Learning Using an Incremental Modular Network Synthesis Approach for Medical Imaging Applications. Appl. Sci..

[18] McKinney S.M., Sieniek M., Godbole V., Godwin J., Antropova N., Ashrafian H., Back T., Chesus M., Corrado G.S., Darzi A. (2020). International evaluation of an AI system for breast cancer screening. Nature.

[19] Rieke N., Hancox J., Li W., Milletari F., Roth H.R., Albarqouni S., Bakas S., Galtier M.N., Landman B.A., Maier-Hein K. (2020). The future of digital health with federated learning. NPJ Digit. Med..

[20] Khan A., Sohail A., Zahoora U., Qureshi A.S. (2020). A survey of the recent architectures of deep convolutional neural networks. Artif. Intell. Rev..

[21] Renjith V.S., Hency Jose P.S. A Noninvasive Approach Using Multi-tier Deep Learning Classifier for the Detection and Classification of Breast Neoplasm Based on the Staging of Tumor Growth. Proceedings of the 2020 International Conference on Decision Aid Sciences and Application (DASA).

[22] Krizhevsky A., Sutskever I., Hinton G.E. (2012). Imagenet classification with deep convolutional neural networks. Adv. Neural Inf. Process. Syst..

[23] Salama W.M., Aly M.H. (2021). Deep learning in mammography images segmentation and classification: Automated CNN approach. Alex. Eng. J..

[24] Szegedy C., Liu W., Jia Y., Sermanet P., Reed S., Anguelov D., Erhan D., Vanhoucke V., Rabinovich A. Going deeper with convolutions. Proceedings of the IEEE Conference on Computer Vision and Pattern Recognition.

[25] Szegedy C., Vanhoucke V., Ioffe S., Shlens J., Wojna Z. Rethinking the inception architecture for computer vision. Proceedings of the IEEE Conference on Computer Vision and Pattern Recognition.

[26] Huang G., Liu Z., Van Der Maaten L., Weinberger K.Q. Densely connected convolutional networks. Proceedings of the IEEE Conference on Computer Vision and Pattern Recognition.

[27] He K., Zhang X., Ren S., Sun J. Deep residual learning for image recognition. Proceedings of the IEEE Conference on Computer Vision and Pattern Recognition.

[28] Simonyan K., Zisserman A. (2014). Very deep convolutional networks for large-scale image recognition. arXiv.

[29] Sandler M., Howard A., Zhu M., Zhmoginov A., Chen L.C. Mobilenetv2: Inverted residuals and linear bottlenecks. Proceedings of the IEEE Conference on Computer Vision and Pattern Recognition, Salt Lake City.

[30] Ronneberger O., Fischer P., Brox T. U-net: Convolutional networks for biomedical image segmentation. Proceedings of the International Conference on Medical image Computing and Computer-Assisted Intervention.

[31] Baccouche A., Garcia-Zapirain B., Zheng Y., Elmaghraby A.S. (2022). Early Detection and Classification of Abnormality in Prior Mammograms using Image-to-Image Translation and YOLO techniques. Comput. Methods Programs Biomed..

[32] Redmon J., Divvala S., Girshick R., Farhadi A. You only look once: Unified, real-time object detection. Proceedings of the IEEE Conference on Computer Vision and Pattern Recognition.

[33] Zhu J.Y., Park T., Isola P., Efros A.A. Unpaired image-to-image translation using cycle-consistent adversarial networks. Proceedings of the IEEE Conference on Computer Vision and Pattern Recognition.

[34] Isola P., Zhu J.Y., Zhou T., Efros A.A. Image-to-image translation with conditional adversarial networks. Proceedings of the IEEE Conference on Computer Vision and Pattern Recognition.

[35] Mobark N., Hamad S., Rida S. (2022). CoroNet: Deep Neural Network-Based End-to-End Training for Breast Cancer Diagnosis. Appl. Sci..

[36] Chollet F. Xception: Deep learning with depthwise separable convolutions. Proceedings of the IEEE Conference on Computer Vision and Pattern Recognition.

[37] Shen T., Gou C., Wang J., Wang F.Y. (2019). Simultaneous segmentation and classification of mass region from mammograms using a mixed-supervision guided deep model. IEEE Signal Process. Lett..

[38] Badrinarayanan V., Kendall A., Cipolla R. (2017). Segnet: A deep convolutional encoder-decoder architecture for image segmentation. IEEE Trans. Pattern Anal. Mach. Intell..

[39] Zhang C., Zhao J., Niu J., Li D. (2020). New convolutional neural network model for screening and diagnosis of mammograms. PLoS ONE.

[40] Zheng J., Lin D., Gao Z., Wang S., He M., Fan J. (2020). Deep learning assisted efficient AdaBoost algorithm for breast cancer detection and early diagnosis. IEEE Access.

[41] Agnes S.A., Anitha J., Pandian S., Peter J.D. (2020). Classification of mammogram images using multiscale all convolutional neural network (MA-CNN). J. Med. Syst..

[42] Sha Z., Hu L., Rouyendegh B.D. (2020). Deep learning and optimization algorithms for automatic breast cancer detection. Int. J. Imaging Syst. Technol..

[43] Ewees A.A., Abd Elaziz M., Houssein E.H. (2018). Improved grasshopper optimization algorithm using opposition-based learning. Expert Syst. Appl..

[44] Clark K., Vendt B., Smith K., Freymann J., Kirby J., Koppel P., Moore S., Phillips S., Maffitt D., Pringle M. (2013). The Cancer Imaging Archive (TCIA): Maintaining and operating a public information repository. J. Digit. Imaging.

[45] Makantasis K., Karantzalos K., Doulamis A., Doulamis N. Deep supervised learning for hyperspectral data classification through convolutional neural networks. Proceedings of the 2015 IEEE International Geoscience and Remote Sensing Symposium (IGARSS).

[46] Makantasis K., Voulodimos A., Doulamis A., Doulamis N., Georgoulas I. Hyperspectral image classification with tensor-based rank-R learning models. Proceedings of the 2019 IEEE International Conference on Image Processing (ICIP).

[47] Makantasis K., Georgogiannis A., Voulodimos A., Georgoulas I., Doulamis A., Doulamis N. (2021). Rank-r fnn: A tensor-based learning model for high-order data classification. IEEE Access.

[48] Kolda T.G., Bader B.W. (2009). Tensor decompositions and applications. SIAM Rev..

[49] LeCun Y., Bengio Y., Hinton G. (2015). Deep learning. Nature.

[50] Kingma D.P., Ba J. (2014). Adam: A method for stochastic optimization. arXiv.

[51] Moreira I.C., Amaral I., Domingues I., Cardoso A., Cardoso M.J., Cardoso J.S. (2012). Inbreast: Toward a full-field digital mammographic database. Acad. Radiol..

[52] Desai M., Shah M. (2021). An anatomization on breast cancer detection and diagnosis employing multi-layer perceptron neural network (MLP) and Convolutional neural network (CNN). Clin. eHealth.

[53] Mohapatra S., Muduly S., Mohanty S., Ravindra J.V.R., Mohanty S.N. (2022). Evaluation of deep learning models for detecting breast cancer using histopathological mammograms Images. Sustain. Oper. Comput..

[54] Han Z., Jian M., Wang G.G. (2022). ConvUNeXt: An efficient convolution neural network for medical image segmentation. Knowl. Based Syst..

[55] Saxena S., Shukla S., Gyanchandani M. (2020). Pre-trained convolutional neural networks as feature extractors for diagnosis of breast cancer using histopathology. Int. J. Imaging Syst. Technol..

[56] Iandola F.N., Han S., Moskewicz M.W., Ashraf K., Dally W.J., Keutzer K. (2016). SqueezeNet: AlexNet-level accuracy with 50× fewer parameters and <0.5 MB model size. arXiv.

